# Gold Nanoparticles Functionalized with Peptides for Specific Affinity Aggregation Assays of Estrogen Receptors and Their Agonists

**DOI:** 10.3390/s120404952

**Published:** 2012-04-18

**Authors:** Yoshiyuki Takatsuji, Shinya Ikeno, Tetsuya Haruyama

**Affiliations:** Department of Biological Functions and Engineering, Kyushu Institute of Technology, Kitakyushu Science and Research Park, Kitakyushu, Fukuoka 808-0196, Japan; E-Mails: takatsuji-yoshiyuki@edu.life.kyutech.ac.jp (Y.T.); ikeno@life.kyutech.ac.jp (S.I.)

**Keywords:** estrogen receptor, gold nanoparticles, high-throughput screening, SRC1 peptide

## Abstract

Nuclear receptors regulate the transcription of genes and various functions such as development, differentiation, homeostasis, and behavior by formation of complexes with ligand and co-activator. Recent findings have shown that agonists of a ligand may have a toxic effect on cellular/tissular function through improper activation of nuclear receptors. In this study, a simple assay system of hetero-complexes of three different molecules (estrogen receptor, ligand, and co-activator peptide) has been developed. This assay system employs functionalized gold nanoparticles (GNPs: 15 nm in diameter). The surfaces of the GNPs were modified by a 12- or 20-amino-acid peptide that contains the sequence of co-activator for activating nuclear receptor by an agonist ligand. Owing to the affinity of the peptide, the functionalized GNPs aggregate faster when the nuclear receptor and the agonist ligand are also present. The aggregation of GNPs can be identified by shifts in adsorption spectrum, which give information about the specificity of agonist ligands. Similarly, this spectrum shift can measure concentration of known agonist ligand. This simple agonist screening will be employed as high through-put analysis (HTA) in the discovery of drugs that act through nuclear receptors.

## 1. Introduction

The development of clinical drugs is also known as drug discovery because most of the lead substances have been found from either natural sources or huge collections of compounds. Finding a lead compound that can be a starting compound for subsequent screening is generally a time-consuming process. The first step of the investigation is key for the successful development of a clinical drug [1]. Hence, high-through-put screening (HTS) in the drug discovery process could be an effective and time-saving procedure for the successful identification of lead-substances because of its efficiency and low-cost for the development of clinical drugs.

In recent years, nuclear receptors have received much attention as molecular targets of clinical drugs being developed against human diseases. Nuclear receptors are transcription factors that mediate the expression of hormones-responsive genes [2,3]. The transcriptional responses depend on the specific type of nuclear receptor, and they play important roles in embryonic development, differentiation, reproduction, and metabolic homeostasis [4]. As two examples, estrogen receptor (ER) and androgen receptor (AR) are well-known nuclear receptors that are strongly involved in breast cancer and prostate cancer, respectively [5,6]. Tamoxifen is well known as an antagonist of ER, and is currently used for the treatment of ER-positive breast cancer [5]. Peroxisome proliferator-activated receptors (PPARs) are directly implicated in lipid transport and metabolism [7]. In particular, PPAR λ agonists are used for their potent antidiabetic effects. In addition, the thiazolidinediones are a class of medicines used in the treatment of diabetes mellitus type 2 [8].

In the recent development of ligand-based drugs, *in vitro* and *in vivo* screening methods have been utilized for the identification of lead-substances. The simplest assays are radio-ligand competition binding assays [9]. These assays can only confirm the binding affinity between a ligand and a nuclear receptor. However, the assay cannot identify ligand characteristics, such as agonist/antagonist activity. In order to evaluate a ligand's activity, various types of methods have been developed. In other words, it is expected that HTS of various NR are developed.

For example, surface plasmon spectroscopy (SPR) and quartz crystal microbalance (QCM) have been well used for affinity sensing of biomolecules in recent years [10,11]. By employing these methods, it is possible to identify a ligand's mode of action. However, these approaches are difficult to utilize for HTS to analyze many samples simultaneously.

Alternatively, reporter gene assays based on stably transfected cell lines are a popular type of *in vivo* assay to evaluate transcriptional activity [12]. This method provides the most specific and responsive means to screen substances for potential activity. However, it is not appropriate for HTS for the identification of lead-substances in drug discovery, because the assay requires more time for evaluation than the above *in vitro* methods.

In this study, we have focused on gold nanoparticles (GNPs) as a signal transducer that enables the affinity interaction between a nuclear receptor and agonist ligands to be detected by shifts in the absorption spectrum. GNPs have unique optical, electrical, and magnetic properties [13]. In particular, the optical spectra of GNPs show a localized surface plasmon band in the region of 520–550 nm. The absorption spectrum of GNPs changes drastically when several particles aggregated [14]. Various types of GNPs based sensors (DNA, antibody, polymer) [15–17] have been developed to take advantage of this property.

In order to discover agonists of nuclear receptors, co-activator proteins have been utilized to analyze the affinity of ligand-activated receptors. Steroid-receptor co-activator-1 (SRC1) is a ligand-inducible transcription factor of the steroid-hormone receptor superfamily. Ligand-activated steroid-hormone receptor forms a complex with SRC-1, and the complex enhances transcriptional activity. LXXLL motifs of co-activators are known to be essential for interaction with ligand-activated nuclear receptors [18].

In this manuscript, we report and discuss a smart assay method using functionalized (molecularly modified) GNPs for ligand screening of human estrogen receptor alpha subtype (hERα). A synthetic peptide containing the LXXLL motif of SRC-1 can be employed as a molecular-recognition element of ligand-activated nuclear receptors. This colloidal sensor was constructed to utilize a modified SRC-1 peptide on the GNP surface for measurement purposes. When ligand-activated hERα forms complexes with functional GNPs, the absorption spectrum of the solution is changed by a decrease of the colloidal stability in the solvent. In this study, we have developed and demonstrated the utilization of this phenomenon for a colorimetric biosensor, and then discussed its application for HTS of nuclear receptor ligands in drug discovery.

## 2. Experimental Section

### 2.1. Chemicals

Gold nanoparticles (15 nm in diameter, 0.0065 wt%) were purchased from Tanaka Kikinzoku Kogyo K.K. (Tokyo, Japan). Purified recombinant human estrogen receptors (hERα), 17β-estradiol, and tamoxifen citrate were purchased from Wako Pure Chemical Industries, Ltd. (Osaka, Japan). Methoxytrityl-S-dPEG_4_ acid was purchased from Quanta Biodesign (Boston, MA, USA).

### 2.2. Synthesis of Functional Peptides

Functional peptide-1 (FP1: its sequence is shown in Table 1) was synthesized by a solid-phase method using fluorenemethyloxycarbonyl (Fmoc) chemistry starting from H-Asp(OTBu)-Trt(2-Cl) resin (0.25 mmol·g^−1^). The removal of the Fmoc group was carried out by treatment with 20% piperidine solution in *N*-methylpyrrolidone (NMP) for 30 min. The condensation reaction was mediated by 2-(1H-benzotriazole-1-yl)-1,1,3,3,-tetramethyluronium hexafluorophosphate (HBTU), 1-hydroxybenzotriazole hydrate and diisopropylethylamine in the same amounts (3 eq., 0.66 mmol) in NMP using a standard protocol [19]. The functional peptide was cleaved from the resin and the protecting group by treatment with a mixture of trifluoroacetic acid, ethanedithiol, thioanisole, water and phenol (80:2.5:5:5:7.5 v/v; 10 mL) for 4 h in an ice bath. After filtration, trifluoroacetic acid (TFA) was evaporated, and the peptides were precipitated by the addition of diethyl ether, centrifuged, resuspended in diethyl ether, and then dried. The desired peptides were purified by high-performance liquid chromatography (HPLC) using an XTerra Prep MS C18 column. The molecular mass of the purified peptide was confirmed by matrix-assisted laser desorption ionization-time of flight-mass spectroscopy (MALDI TOFMS). Similarly, functional peptide-2 (FP2: This sequence is shown in Table 1) was synthesized by a solid-phase method and checked the molecular mass using LCMS.

### 2.3. Affinity Assay Between Synthesized Functional Peptide and hERα on Gold Plate

The functional peptides were modified on a gold plate through thiol for 1 h. After the modification, the functional peptides were confirmed to have formed complexes with ERα using EnBio RCAS for ERα (Fujikura Kasei Co., Ltd. Tokyo, Japan) [20].

### 2.4. Binding of Modified Functional Peptides with Gold Nanoparticles

FP1 at 5 μg/mL and FP2 750 μg/mL were prepared with 10 mM citrate buffer (pH 6.0). These solutions were mixed in a 1:1 ratio with GNPs (15 nm) and incubated for 1 h. To remove free functional peptides, the particle solutions were centrifuged (∼14,000 g) for 20 min and the supernatant was removed and replaced with hERα reaction buffer (10 mM HEPES (pH 7.4), 200 mM NaCl, 10% glycerol, 0.05% Tween 20). Characterization of particle size and zeta potential were assayed by Zetasizer Nano ZS (MALVERN, Malvern, UK).

### 2.5. UV-Vis Spectra of GNP Measurement

GNPs were added to 45 nM of hERα and with or without ligand (E2 or tamoxifen).The mix solution incubated at 25 °C. Then the absorbance spectrums of solution were measured using Ultrospec 3300 pro (GE Healthcare, Little Chalfont, UK) at each time point.

## 3. Results and Discussion

**Figure 1.**
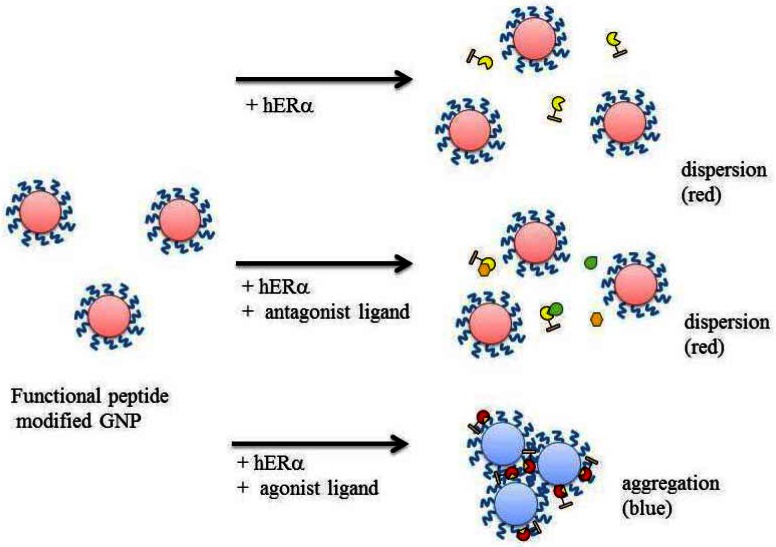
Schematic illustration of detection of ligand activity using modified functional peptide-associated GNPs.

Figure 1 shows a schematic view of the agonist ligand assay for a nuclear receptor with GNPs. Functional peptide-modified GNPs (GNP-FP) are dispersed in aqueous solution by the action of the modified functional peptides. In this study, hERα is employed as a model target molecule (analyte). GNP-FP is mixed with hERα activated by an agonistic ligand in advance. Then, the hERα forms a complex with the functional peptide on the GNP surface. As a result, GNP-FP aggregates and its absorption spectrum is changed. On the other hand, the antagonistic ligand cannot activate hERα and thus GNP-FP does not aggregate in this case. In addition, its absorption spectrum is not changed because GNP-FP cannot form a complex with antagonistic ligand binding hERα.

As shown in Table 1, FP1 and FP2 were designed on the basis of SRC-1 NR box II within the LXXLL motif, which can form a specific complex with activated hERα[18]. FP1 consists of 20 amino acid peptide that can bind covalently with gold through the N-terminal cysteine in the peptide. FP2 is composed of the following functional units: (1) polyethylene glycol (PEG) that was introduced in order to suppress unspecific adsorption of hydrophobic peptide to the nanoparticle surface (thiol was also tagged to the N-terminal of the PEG for binding to GNPs); and (2) the 12-mer peptide, which is also designed on SRC-1 NR box II to form a specific complex with hERα activated by agonist ligand. Above the design concept of functional peptide, we expect that FP2 is superior to FP1 as a molecular-recognition element of functionalized GNPs.

**Figure 2.**
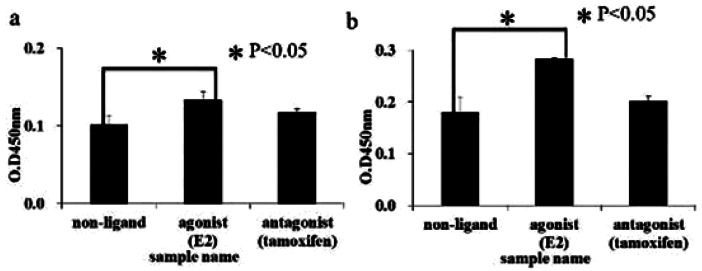
Confirmation of the presence of complex between synthesized functional peptide and hERα on gold plate (n = 3): (**a**) modification of FP1; (**b**) modification of FP2.

First, we investigated complex formation between functional peptide and hERα with or without ligand, as shown in Figure 2. This figure shows the affinity assay of synthesized peptides with hERα activated by agonist ligand using the affinity assay kit. The agonist ligand (E2) sample shows a higher level of optical density than the other samples [with no ligand or antagonist ligand (tamoxifen)] and shows a significant difference in this regard compared with the other samples. Accordingly, the synthesized functional peptides were expected to have ligand selectivity for hERα and form a specific complex with estrogen receptor.

**Figure 3.**
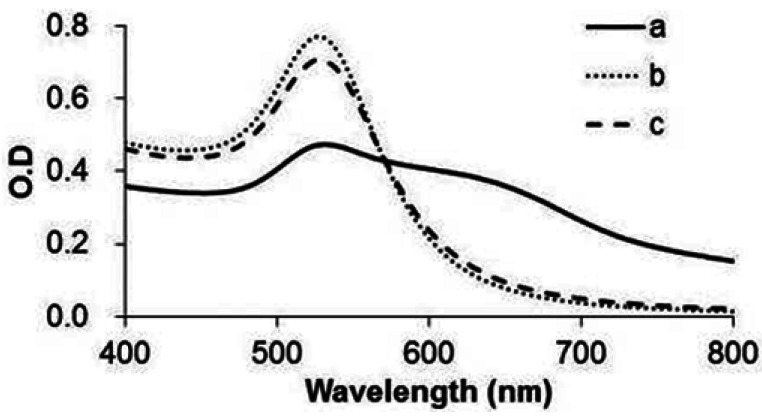
UV-Vis spectra of GNPs in hERα reaction buffer: a—control (without modification of functional peptides); b—modification of FP1; and c—modification of FP2.

Functionalized GNPs can be dispersed in hERα reaction buffer owing to modified functional peptide (Figure 3). When bare GNPs in citrate buffer is changed to in hERα reaction buffer, the GNPs aggregate. But GNP-FP1 and 2 can remain dispersed in hERα reaction buffer. Moreover, average size and zeta potential of functionalized GNPs are 21.6 nm and −9.53 mV. This result indicated the functional peptides on the GNP surface have a negative charge in the buffer and induce dispersion of GNP in the buffer. The difference in the absorption spectrum between GNP-FP1 and 2 depends on the electric permittivity on the GNP surface [21].

**Figure 4.**
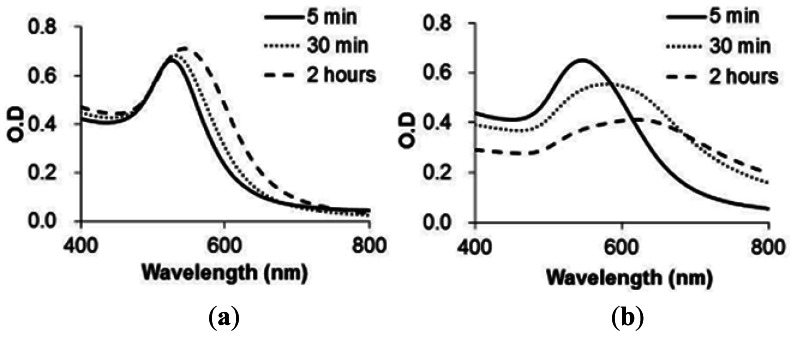
UV-Vis Spectra of functional GNPs at 5 min, 30 min and 2 h after addition of hERα and E2; (**a**) GNP-FP1; (**b**) GNP-FP2.

Figure 4 shows the absorption spectra of GNP-FP1 and 2 with hERα activated by agonist ligand (E2) for each reaction time. Both spectra were shifted to the right by mixture with activated hERα over time. The results showed that the spectrum of GNP-FP2 (Figure 4(a)) changed faster than that of GNP-FP1 (Figure 4(b)). This indicates that FP2 more efficiently formed a complex with hERα activated by agonist ligand on GNPs than FP1. For GNP-FP1, specific complexes between FP1 and activated hERα were not formed efficiently on GNPs because the hydrophobic sequences of FP1 caused non-specific adsorption to the GNP surface. However, in the case of FP2, PEG of FP2 inhibited non-specific adsorption by hydrophobic sequences. Hence, GNP-FP2 aggregated faster than GNP-FP1 by the specific formation of complexes with hERα activated by agonist ligand.

**Figure 5.**
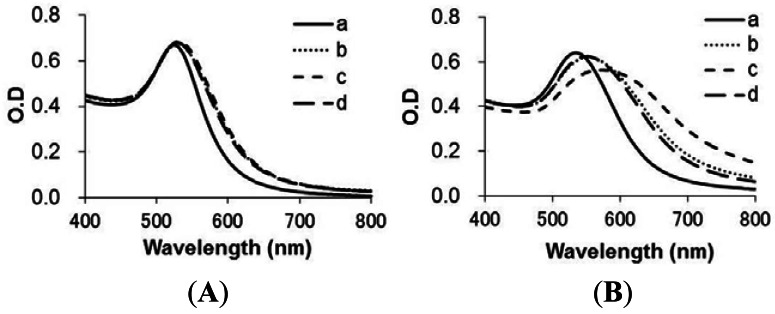
UV-Vis Spectra of GNPs in the presence of (a) control (without hERα and ligand); (b) hERα; (c) hERα and E2; and (d) hERα and tamoxifen at 30 min (addition of substances to be examined). (**A**) Modification of FP1; (**B**) modification of FP2.

To determine aggregation by the specific formation of complexes with hERα on GNPs in a ligand-dependent manner, we obtained the spectra of GNP-FP1 and 2 at 30 min after the addition of hERα and ligands (Figure 5). In the case of GNP-FP1, the change of spectrum was dependent on ligand at 30 min (Figure 5(A)), but at 2 h (data not shown), the spectrum of GNP-FP1 was shifted to the right in the case of E2 compared with the other ligands. On the other hand, in the case of GNP-FP2, the spectra were already shifted to the right compared to the other ligands at 30 min already (Figure 5(B)). Both results show that the sample of agonist ligand (E2) causes a larger change in the spectrum than the other ligand samples. These results indicate that GNP-FP1 and 2 can be used to estimate ERα activity in a ligand-dependent manner. Namely, it is possible for agonist activity to be measured by determining the rate of aggregation. In addition, GNP-FP2 can be used to determine the agonist activity of hERα faster than GNP-FP1.

**Figure 6.**
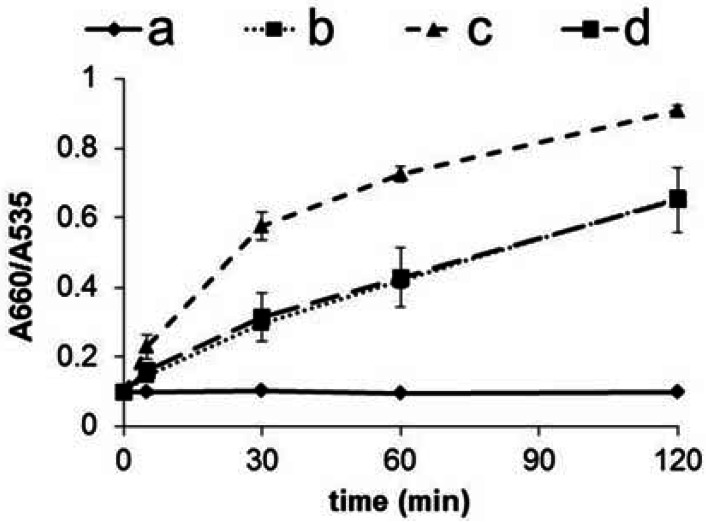
Aggregation kinetics of GNP-FP2 in the presence of a—control (without hERα and ligand); b—hERα; c—hERα and E2; and d—hERα and tamoxifen.

To easily estimate ligand activity of hERα from spectrum data, we defined the ratio of absorbances at 660 nm/535 nm as the sensor response. 535 nm is wavelength of adsorption maximum corresponding to both GNP-FP1 and 2. For its part, 660 nm is value of maximum in differential spectrum between aggregation of before and after. The sensor response is recorded over time, which enables estimation of the ligand activity (Figure 6). From sensor response data, E2 (agonist ligand) was shown to enable faster aggregation of GNP than the other samples. In contrast, tamoxifen (antagonist ligand) produced a result similar to the case without a ligand because tamoxifen bound to ERα prevents formation of a complex with co-activator. Briefly, the sensor responses indicate that the specific formation of a complex on the GNP surface is dependent on the ligand and can be used to estimate agonist activity of the ligand.

**Figure 7.**
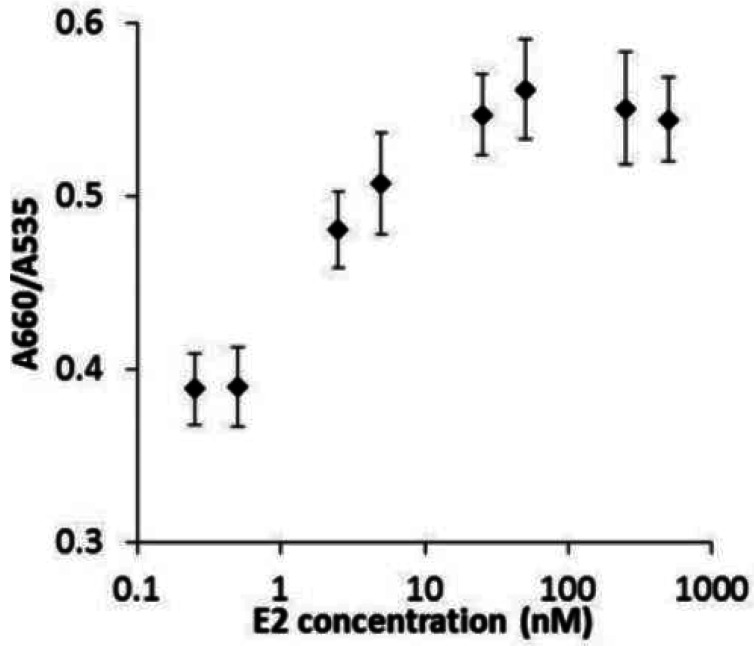
Degree of aggregation in term of E2 concentration with GNP-FP2 (n = 3).

Using GNP-FP2, we attempted to determine E2 concentrations by the sensor response, which was measured 30 min after the reaction of activated hERα with E2 (Figure 7). Figure 7 shows relation between E2 concentration and sensor response. The plots clearly showed sigmoid curve. Below 0.5 nM, the sensor response is not enough to determine E2 concentration. However, between 0.5 nM to 25 nM, the sensor response is linear against E2 concentration. In the higher concentration than 25 nM, the response reachs a plateau. This finding showed why hERα concentration is constant 45 nM in every concentration of the ligand. Therefore, the plateau of response is between approximately 25 nM to 50 nM. because hERα and E2 were reactive at a ratio of 1:1.

## 4. Conclusions

In this study, using GNP-FP, we succeeded in detecting specific formation of complexes with hERα, functional peptide and ligands. The three-molecule hetero-complex assay is based on the *in situ* function of hERα. The system can recognize ligands that are inappropriate for drug development of nuclear receptors by a simple method. It can be systemized with HTS in good reproducibility. The advantages of this method are as follows: (1) hERα and ligands do not require labeling; and (2) the substance to be examined can be screened as an agonist in 30 min. Given these advantages, this method is expected to be applicable for the first stage of screening in drug discovery. In this study, hERα was used as a model for agonistic ligand screening of nuclear receptor. However, it is possible to expand this system for other nuclear receptors. Moreover, it is expected that the method can be used to estimate the level of agonist activity on a nuclear receptor and to determine the concentration of agonist ligand by sensor response.

## Supplementary Material



## Figures and Tables

**Table 1.** Designed sequences of functional peptides.

**Peptides**	**Designed Sequences**

**Functional peptide 1(FP1)**	CLTERHKILHRLLQEGSPSD
**Functional peptide 2(FP2)**	Thiol-PEG_4_-RHKILHRLLQED
