# Supplementary material for: Gold Nanoparticles Functionalized with Peptides for Specific Affinity Aggregation Assays of Estrogen Receptors and Their Agonists
Source: Sensors (Basel). 2012 Apr 18;12(4):4952–61. doi: 10.3390/s120404952 (PMC3355452; doi:10.3390/s120404952)
Supplement: Supplementary file 1 [file sensors-12-04952-s001.pdf]

Supplemental Information

Gold Nanoparticles Functionalized with Peptides for Specific Affinity Aggregation Assays of Estrogen Receptors and Their Agonists

Yoshiyuki Takatsuji, Shinya Ikeno and Tetsuya Haruyama *

Department of Biological Functions and Engineering, Kyushu Institute of Technology, Kitakyushu Science and Research Park, Kitakyushu, Fukuoka 808-0196, Japan;
E-Mails: takatsuji-yoshiyuki@edu.life.kyutech.ac.jp (Y.T.); ikeno@life.kyutech.ac.jp (S.I.)

* Author to whom correspondence should be addressed; E-Mail: haruyama@life.kyutech.ac.jp.

Received: 26 March 2012; in revised form: 9 April 2012 / Accepted: 16 April 2012 /
Published: 18 April 2012

Synthesized FP1 were confirmed by HPCL and MALDITOF MS (Figures S1 and S2).

Figure S1. HPLC analysis of synthesized FP1 using XTerra MS C18 (4.6 × 150 mm × 3 μm) with UV detect at 220 nm (Linear gradients between 0% CH_3CN and 100% H_2O to 75% CH_3CN and 25% H_2O in 15 min at a flow rate of 2 mL/min).

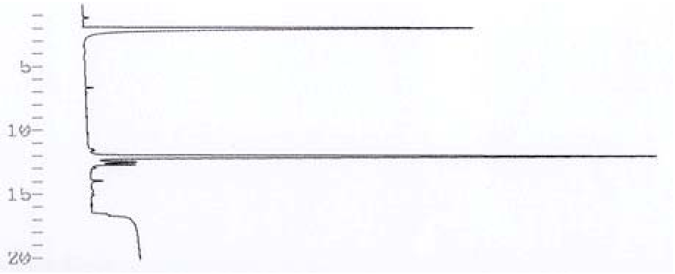

Figure S2. MALDI TOFMS spectrum of synthesized FP1 with α-cyano-4-hydroxycinnamic acid.

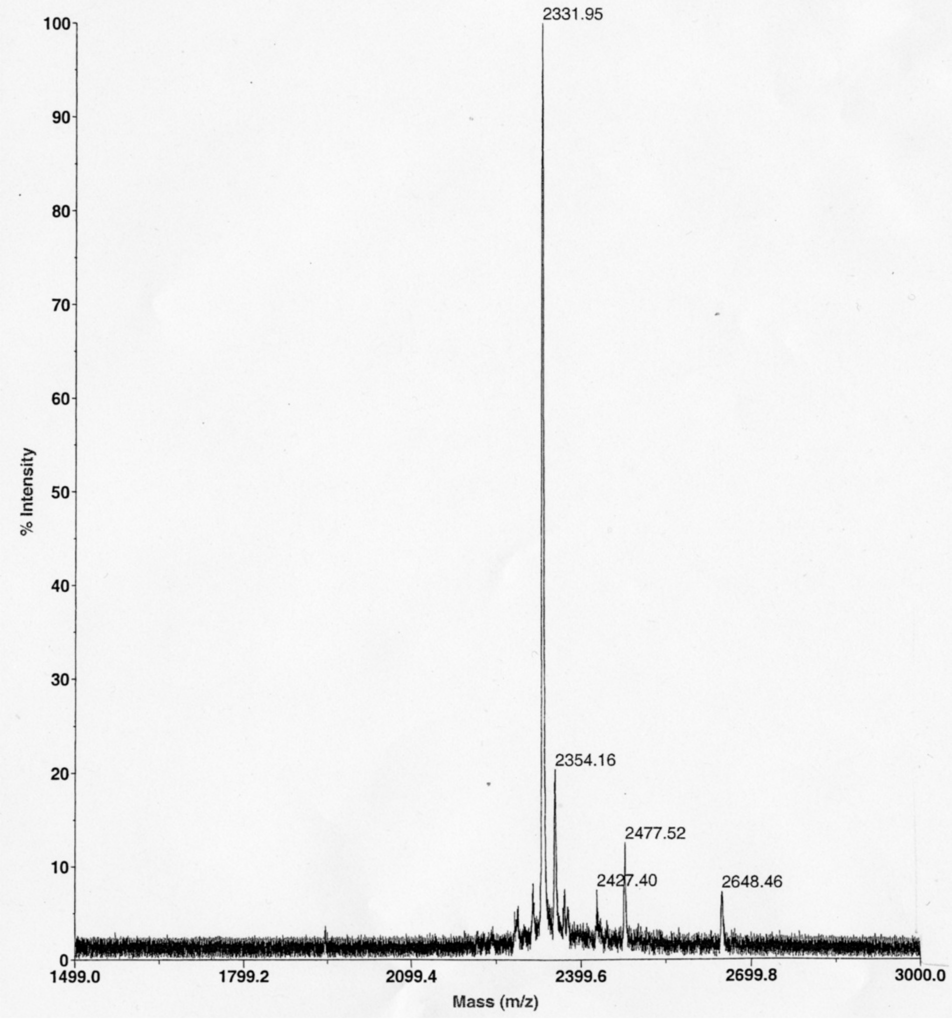

Synthesized FP2 were confirmed by LCMS (Figures S3 and S4).

Figure S3. HPLC analysis of synthesized FP2 using XTerra MS C18 (4.6 × 150 mm × 3 μm) with UV detect at 220 nm (Linear gradients between 0% CH_3CN and 100% H_2O to 40% CH_3CN and 60% H_2O in 15 min at a flow rate of 2 mL/min).

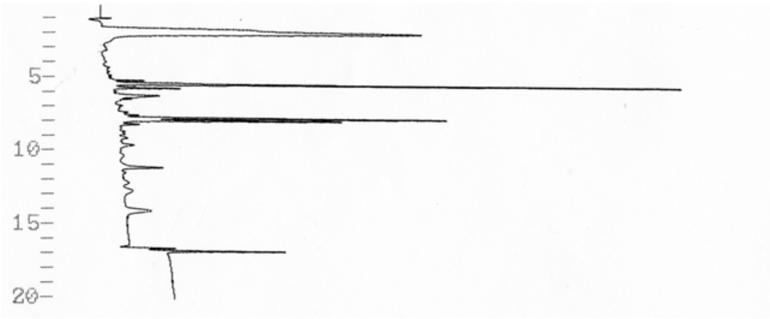

Figure S4. LCMS spectrum of synthesized FP2 with API 150EX (AB SCIEX).

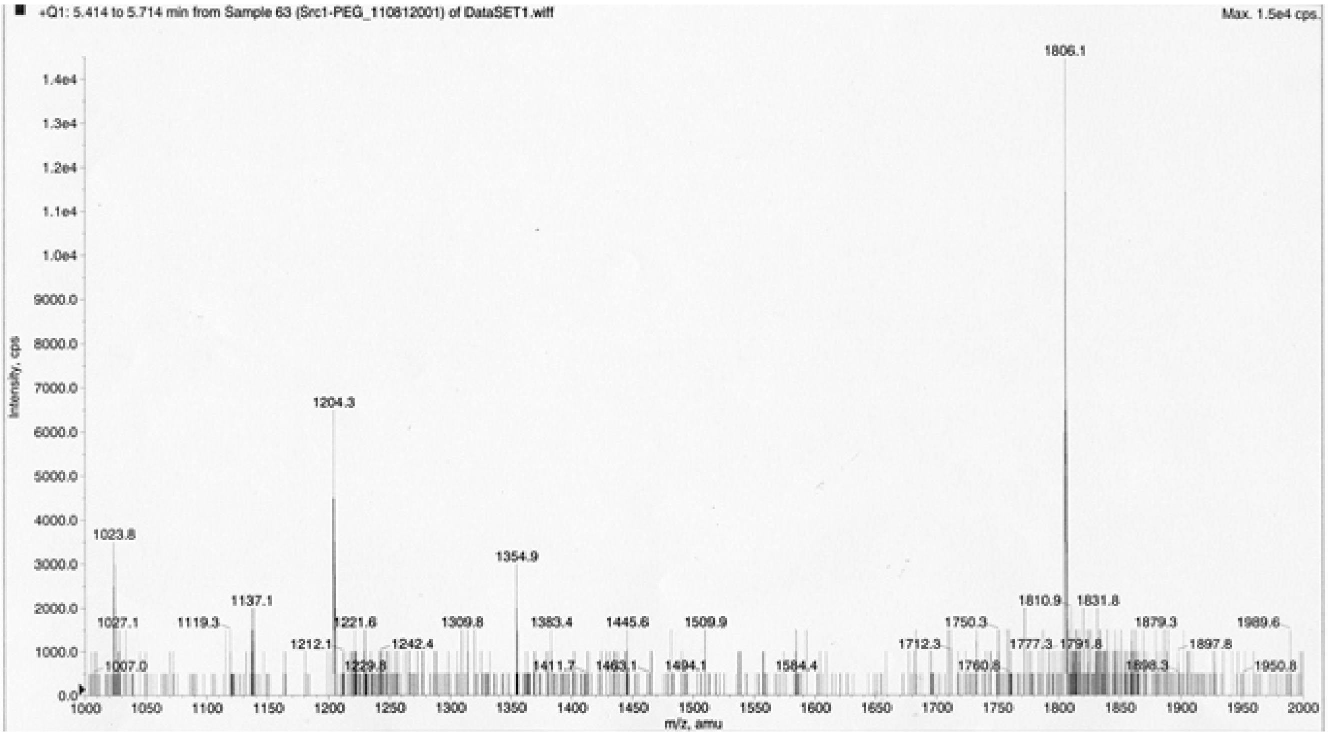

© 2012 by the authors; licensee MDPI, Basel, Switzerland. This article is an open access article distributed under the terms and conditions of the Creative Commons Attribution license (http://creativecommons.org/licenses/by/3.0/).
